# Hydrogen-Induced Cracking Susceptibility of API 5L X100 Steel Welded Joint

**DOI:** 10.3390/ma19143114

**Published:** 2026-07-20

**Authors:** Chunyan Yan, Lingchuan Zhou, Qianwen Zhou, Tiancheng Yao, Xinyi Liu, Qiqing Lu

**Affiliations:** 1 College of Materials Science and Engineering, Hohai University, Changzhou 213022, China; 231325020007@hhu.edu.cn (L.Z.); 251616030031@hhu.edu.cn (T.Y.); 2261410203@hhu.edu.cn (X.L.); luqiqing@hhu.edu.cn (Q.L.); 2 School of Materials Science and Engineering, Zhejiang University, Hangzhou 310013, China; 12326095@zju.edu.cn

**Keywords:** steel, hydrogen-induced cracking, hydrogen permeation, slow strain rate tensile test, welding

## Abstract

Hydrogen-induced cracking (HIC) is a possible failure mode of high-strength pipeline steel welded joints. The HIC susceptibility of shielded metal arc-welded joint of X100 pipeline steel was investigated using the slow strain rate tensile test (SSRT) under current densities of 2 mA/cm^2^, 5 mA/cm^2^, 10 mA/cm^2^, 20 mA/cm^2^, and 30 mA/cm^2^. Hydrogen diffusion behavior, distribution of microstructures, and inclusions in the welded joint were also surveyed and characterized. The SSRT results indicated that the welded joint exhibited a higher hydrogen embrittlement index than the base metal (BM) under the same current density, showing a higher HIC sensitivity than the BM. The weld metal (WM) showed a lower hydrogen diffusivity (5.38 × 10^−5^ cm^2^·s^−1^) than the BM (6.23 × 10^−5^ cm^2^·s^−1^). The martensite–austenite (M-A) constituent area fraction in the WM was higher than that in the coarse-grained heat-affected zone (CGHAZ), fine-grained HAZ (FGHAZ), and BM. The inclusion amount in the WM was larger than that in the BM. In addition, the high HIC vulnerability of the X100 steel welded joint was also associated with a coarse grain size in the WM.

## 1. Introduction

Given that the hydrogen absorption happened in manufacturing processes (acid pickling and welding) and operation processes, hydrogen-induced failure of oil–gas pipelines is posing a serious threat to the operating integrity of pipelines designed with higher strength and operational pressure [1,2,3,4]. It has been reported that about 1/4 of the failure accidents in petroleum equipment can be attributed to hydrogen-associated damage [5,6,7]. With the demand for developing pipeline steels of X90/X100 grade to fulfill higher transport capacity at lower cost, hydrogen-induced damage problems have been prompted and need to be understood based on a general conception that higher HIC susceptibility is associated with higher strength for steels [5,6,7].

Numerous studies have been conducted on HIC susceptibility of pipeline steels, and hydrogen-induced ductility loss was observed in pipeline steels with various strength grades. Boukortt [8] studied the variation in X52 steel mechanical properties considering the influence of HIC. They observed that HIC had a faint effect on yield strength and ultimate strength, but had a detrimental effect on toughness and plasticity. Ning [9] investigated the effect of hydrogen on the fatigue properties of X65 steel and its weld metal under different hydrogen partial pressures. It was found that the weld metal was more sensitive to the hydrogen effect than the base metal due to the microstructure with texture and heterogeneity. Entezari [10] reported that X80 pipeline steel with fewer non-metallic inclusions showed higher HIC resistance and hydrogen diffusivity than X70 pipeline steel. Vishal [11] investigated the effect of inclusions on the HIC sensitivity of X65 pipeline steel and found that the cracks were mainly caused by the presence of a majority of inclusions mixed in Al–Si–O. However, Hu [12] investigated the inclusions in steels treated with Al–Ti deoxidization, and concluded that the trapping efficiency of the inclusions descended in the order of MnS, Al_2_O_3_, MgAl_2_O_4_, and TiN. Huang [13] reported that X120 steel with the microstructures of granular bainite (GB) and M-A constituents was very vulnerable to hydrogen-induced failure. Though numerous investigations maintained that the strength, microstructure, and inclusions were important factors causing HIC, a number of studies gave inconsistent conclusions on the role of the factors. For example, it is generally perceived that elongated MnS inclusions are strong hydrogen traps and most detrimental to HIC [14]. However, Jack [15], Reza [16], and Zhang [17] reported that the Si-enriched and Al-enriched inclusions were the initiation sites for HIC rather than the strip-shaped MnS inclusions. HIC is also greatly influenced by other microstructural factors like grain boundaries, precipitated phases and M-A constituents that affect hydrogen trapping, crack initiation and growth [18,19,20]. Yet research on the effect of microstructures and inclusions on HIC behavior in X100- and X120-grade steel is relatively limited compared to X65–X80-grade steels. Furthermore, most studies focused on the HIC susceptibility of the base metal, while studies of the HIC behavior in welded joints of X100-grade pipeline steel or higher grade are very scarce. Due to the heterogeneity in microstructure and stress, HIC susceptibility of high-strength-steel welded joints can be elevated in hydrogen environments [21,22]. Araújo [23] investigated the HIC susceptibility of X80 steel welded joints using shield metal arc welding (SMAW) and gas tungsten arc welding, and reported that the welded joint with high hardness showed high HIC susceptibility. However, Zhou [24] reported a different result, that the X60 steel welded joint fabricated by rotary friction welding showed a higher HIC resistance than the BM in a 6.3 MPa hydrogen environment. The conflicting conclusions prevent a consistent understanding of HIC susceptibility of high strength steel joints, and the respective effects of the factors remain unclear. Therefore, it is necessary to study the HIC susceptibility of welded joints for higher-strength pipeline steel.

Due to the improvement in thermo-mechanical controlled process (TMCP) and accelerated cooling technology, the X100 steel microstructure is mainly composed of fine bainite/ferrite and M-A constituents, and it is significantly different from the microstructures of the lower-strength-grade steels [25,26]. Moreover, segregation and banded pearlite are barely found in X100 steel. Thus, the mechanism of HIC behavior in X100-grade steel and its welded joint may differ from that of lower-grade steel joints. To resolve these inconsistencies, this work aimed to clarify the correlations between the microstructures, inclusions, M-A constituents and the HIC susceptibility of X100 steel joints. A combination of microstructure characterization, SSRT test, and hydrogen permeation test were carried out to investigate the respective effects of the factors on HIC behavior in a 690 MPa-grade X100 steel and its shielded metal arc-welded joint.

## 2. Materials and Methods

### 2.1. Materials and Welding Parameters

The X100 steel plates investigated were produced by a Chinese steel corporation (Baowu Steel Group, Shanghai, China) and prepared with a size of 200 mm × 100 mm × 18.4 mm (thickness). Following the ISO 9692-1:2013 standard [27], the plates were cut into a 60 ° V-groove to ensure full penetration and good sidewall fusion, and then butt-welded using SMAW method with 4.0 mm diameter cellulosic E10018 electrodes produced by an Austrian corporation (Voestalpine Böhler Welding Austria GmbH, Kapfenberg, Austria). The welding machine used was a ZX7-400 direct-current arc welding machine (Huayuan, Chengdu, China). The main chemical compositions of the BM and electrode were tested using an OES 1000 spectrometer (Skyray, Suzhou, China) and presented in Table 1. The ten-pass welding was carried out using a medium-heat input of 22 kJ·cm^−1^ and an inter-pass temperature of 100 °C to prevent cold cracking. The welding current was 175 A and the voltage was 25 V. The multi-pass welding was repeated three times for reproducibility, and the welded plates were dissected to guarantee no visible welding defects in samples for successive tests.

### 2.2. Slow Strain Rate Tensile Test

**Figure 1:**
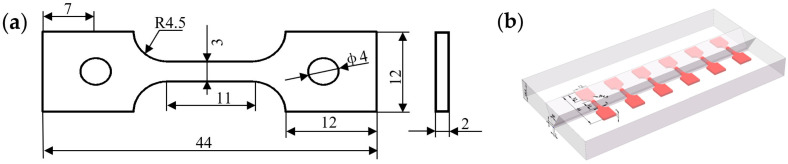
Schematic of SSRT specimens: (**a**) geometry and dimensions, (**b**) specimen positions in the welded joint.

To evaluate the HIC sensitivity of the X100 steel and its welded joint, the SSRT test using a WDML-50 stress corrosion testing machine (Letry, Xi’an, China) was carried out at a strain rate of 1 × 10^−5^ s^−1^. The SSRT test was conducted following the ASTM E8/E8M-25 standard [28], and a minimum of three specimens were tested per condition to ensure repeatability. The gauge length is 11 mm and the total length is 44 mm (Figure 1). The SSRT specimens were cut, ground using 200~1500 grit SiC papers and rinsed using ethanol for the base metal and welded joint, respectively. Using a CS1002 potentiostat/galvanostat (Wuhan Corrtest, Wuhan, China), electrochemical hydrogen charging was performed in a mixed solution of 0.5 mol/L H_2_SO_4_ + 0.5 g/L CH_4_N_2_S under the charging current densities of 2 mA/cm^2^, 5 mA/cm^2^, 10 mA/cm^2^, 20 mA/cm^2^, and 30 mA/cm^2^. The hydrogen charging time for all experimental groups was 4 h. Fracture morphologies of the SSRT specimens were analyzed using a Gemini 300 (ZEISS Ltd., Jena, Germany) scanning electron microscope (SEM). All SSRT specimens of the welded joint were etched with 4% nital reagent to identify the actual fracture positions. The hydrogen embrittlement index *I*_Z_ is often calculated by Equation (1), reported in different studies [10,29].

(1) $$I_{Z}=\frac{Z_{\mathrm{UC}}-Z_{\mathrm{HC}}}{Z_{\mathrm{UC}}}$$

where *Z*_UC_ is the reduction-of-area of uncharged SSRT specimen, and *Z*_HC_ is the reduction-of-area of SSRT specimen performed with hydrogen pre-charging.

### 2.3. Microstructure and Inclusion Characterization

The specimens for microstructure observation were ground, polished and etched using a 4% nital reagent. The M-A constituents in the microstructures were revealed adopting LePera’s reagent (30 mL water + 30 mL Picric acid + 0.30 g Na_2_S_2_O_5_). The microstructure morphology and distribution of the M-A constituents were studied using a ZEISS SEM. The energy dispersive X-ray spectroscopy (EDS) analysis of the inclusions and the vicinal matrix was conducted using a Quanta 250FEG SEM (FEI, New York, NY, USA).

**Figure 2:**
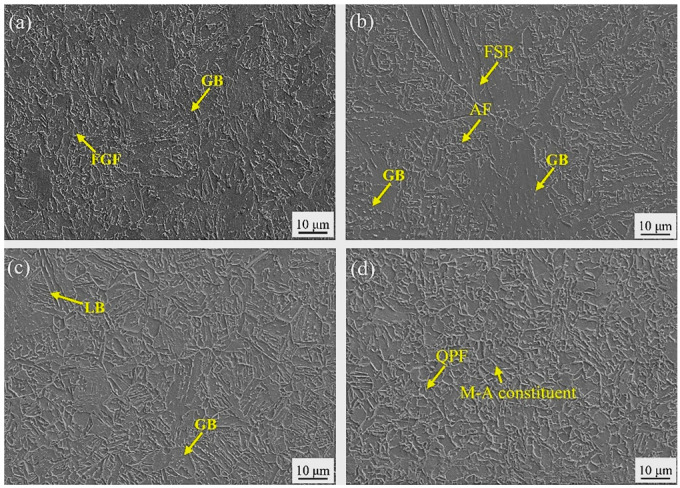
Microstructures in different subzones: (**a**) BM, (**b**) WM, (**c**) CGHAZ, (**d**) FGHAZ.

Figure 2 illustrates the microstructures in the BM, WM, CGHAZ, and FGHAZ. The BM microstructure was comprised of GB and fine-grained ferrite (FGF) which contributed to the high toughness of the steel. The WM microstructure was composed of a small amount of acicular ferrite (AF), ferrite side plate (FSP), and a great amount of GB. The CGHAZ microstructure mainly consisted of GB and LB, while the FGHAZ microstructure consisted of fine quasi-polygonal ferrite (QPF) with a small fraction of M-A constituents.

### 2.4. Hydrogen Permeation Test

**Figure 3:**
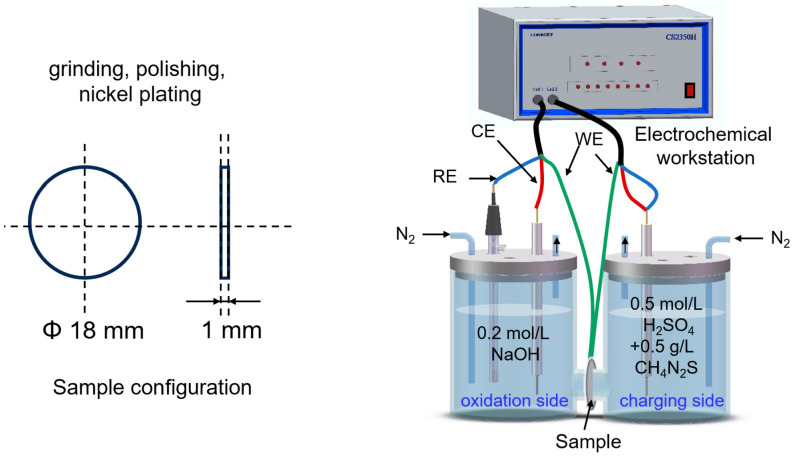
Hydrogen permeation test device.

Based on ASTM G148-97 (2018) [30], the hydrogen permeation test in a dual-cell Devanathan–Stachurski system was carried out to obtain hydrogen diffusion parameters including effective hydrogen diffusivity *D*_eff_, apparent solubility *C*_0_, and number of hydrogen trapping sites *N*_T_, which was reported to characterize HE sensitivity of steels [29], and the device is shown in Figure 3. Samples cut from the BM, WM, and CGHAZ with a size of *Φ*18 mm × 1 mm were ground and polished to 1500 grit. Before permeation, the samples were plated with nickel on one side in a plating solution of 240 g/L NiSO_4_·+ 45 g/L NiCl_2_ + 40 g/L H_3_BO_3_, applying a cathode current density of 4 mA/cm^2^ for 6 min. A mixed solution of 0.5 mol/L H_2_SO_4_ + 0.5 g/L CH_4_N_2_S was adopted for hydrogen charging cell, while a 0.2 mol/L NaOH solution was adopted in the oxidation cell. High-purity nitrogen gas was bubbled into the hydrogen charging cell and oxidation cell to eliminate oxygen dissolved in the solutions. A 300 mV constant voltage was applied for the oxidation side to ensure the oxidization of hydrogen atoms from the hydrogen charging cell to form H^+^. When the background current density dropped below 2 μA/cm^2^ and stayed stable, it was considered that the residual diffusible hydrogen had no effect on the test result, and a 10 mA/cm^2^ current density was applied for the hydrogen charging side. The hydrogen permeation curves (current density–time curves) of the BM, WM, and CGHAZ were recorded and analyzed to calculate hydrogen permeation flux *J*_∞_, effective diffusion coefficient *D*_eff_, hydrogen concentration *C*_0_, and total hydrogen trap density *N*_T_.

In the first hydrogen permeation cycle, hydrogen traps in the specimen consisted of irreversible and reversible hydrogen traps, and the calculated hydrogen trap density was the total hydrogen trap density *N*_T_. In the second hydrogen permeation cycle, only the effect of reversible hydrogen traps was reflected, and the calculated hydrogen trap density was the reversible hydrogen trap density *N*_r_. The irreversible hydrogen trap density *N*_ir_ could be calculated using the obtained *N*_T_ and *N*_r_ [29].

*J*_∞_ can be calculated by Equation (2) using hydrogen current *I*_∞_ [29]:

(2) $$J_{\infty }=\frac{I_{\infty }}{FA}$$

where *F* is Faraday constant (96,500 C/mol) and *A* is the contact area of sample with solution.

*D*_eff_ can be calculated using Equation (3) [29]:

(3) $$D_{\mathrm{eff}}=\frac{d^{2}}{6t_{L}}$$

where *d* is the thickness of tested membrane and *t*_L_ is the lag time at current of *I*_∞_.

*C*_0_ can be calculated using Equation (4) [29]:

(4) $$C_{0}=\frac{J_{\infty }\cdot d}{D_{\mathrm{eff}}}$$

*N*_T_ is the hydrogen trapping sites density obtained using *C*_0_ and *D*_eff_ [29]:

(5) $$N_{T}=\frac{C_{0}}{3}\times (\frac{D_{L}}{D_{\mathrm{eff}}}-1)$$

where *D*_L_ is lattice diffusion coefficient for ferritic steels (1.28 × 10^−4^ cm^2^·s^−1^) [29].

## 3. Results

### 3.1. Microstructural Characterization

**Figure 4:**
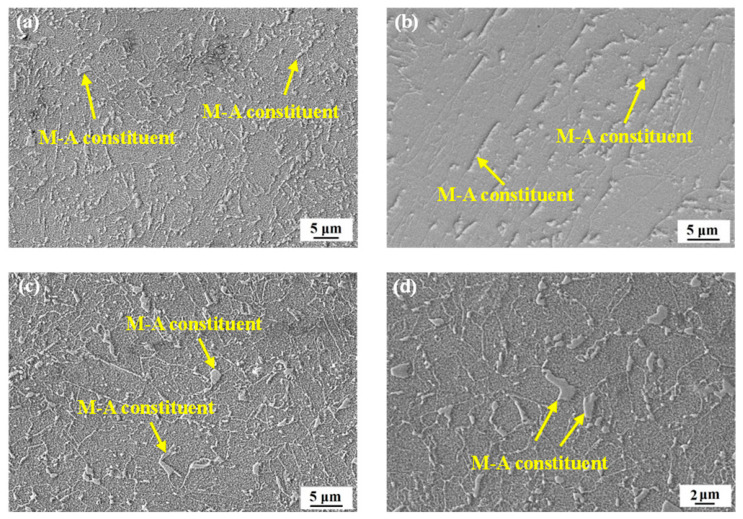
SEM observation of M-A constituents in the welded joint: (**a**) BM, (**b**) WM, (**c**) CGHAZ, (**d**) FGHAZ.

It is reported that M-A constituents are strong hydrogen traps within bainite and are considered as HIC cracking nucleation sites. The hydrogen-induced cracks can nucleate and propagate along or inside the M-A constituents due to its high dislocation density [29]. The etched M-A constituents in four subzones are shown in Figure 4. The M-A constituent fractions in the subzones of the welded joint were studied using the Image Pro Plus 6.0 software based on analysis of at least fifteen SEM morphology images for each subzone. The average values of M-A constituent fractions (Table 2) in the BM, WM, CGHAZ, and FGHAZ were 7.73%, 11.21%, 10.52%, and 9.47%, respectively. The M-A constituent fraction in WM was 1.5 times that in BM.

**Figure 5:**
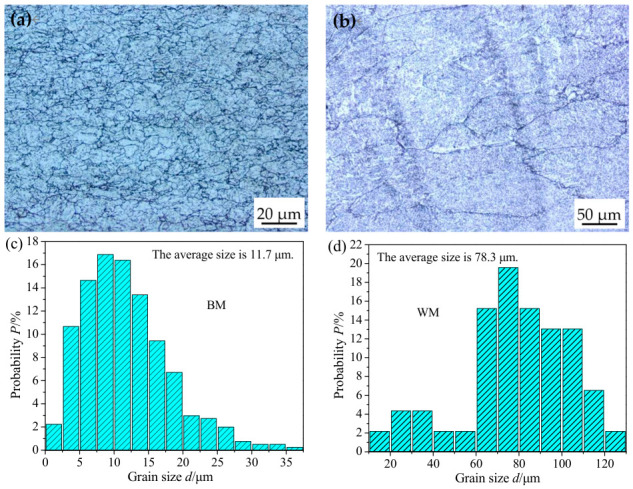
Grain boundaries of and grain size distribution: (**a**) grain boundaries in the BM, (**b**) grain boundaries in the WM, (**c**) grain size distribution in the BM, (**d**) grain size distribution in the WM.

The distribution of grain sizes in the BM and WM based on analysis of twenty images for each zone is shown in Figure 5. The average grain sizes in the BM and WM were about 11.7 μm and 78.3 μm, respectively.

**Figure 6:**
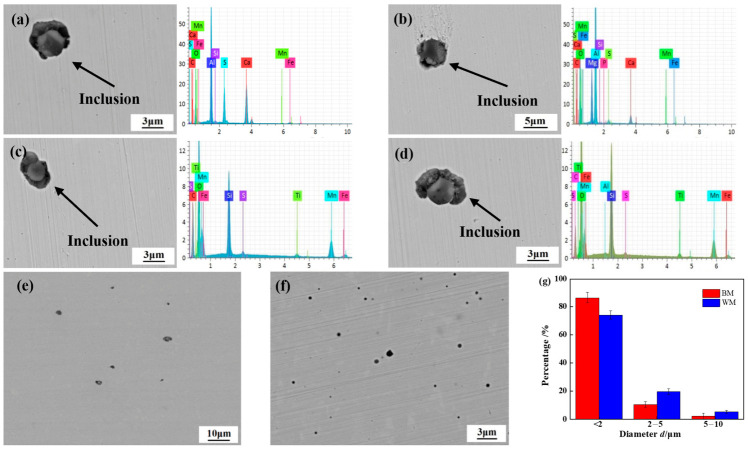
Typical chemical analysis and morphologies of inclusions in the X100 steel (**a**,**b**,**e**) and weld metal (**c**,**d**,**f**), and inclusion size distribution (**g**).

The shapes, dimensions, chemical compositions, and distribution of inclusions in the X100 steel and WM were analyzed and are shown in Figure 6. The inclusions in the BM and WM were basically spherical and could be classified into three types: (1) Ca–Al–O–S inclusion (Figure 6a), which was typical for Al killed X100 steels, (2) Al–Mg–Ca–O inclusions (Figure 6b), and (3) Si–Mn–O inclusions (Figure 6c,d). It is seen from Figure 6e–g that over 70% of the inclusions had diameters below 2 μm. The fraction of large inclusions with size over 2 μm was 24.92% in the WM, higher than that in the BM (12.73%). It is reported that only non-metallic inclusions with size larger than 2 μm could be effective in elevating the HIC susceptibility [3]. The inclusion amounts in the BM and WM were analyzed using 50 SEM images, and the results with relative standard deviation in the range of ±5% showed that inclusion amount in the WM (190.73/mm^2^) was larger than that in the BM (11.02/mm^2^). However, we found no cracking occurred at inclusions enriched in MnS, and this conclusion is consistent with studies conducted by Jack [15].

### 3.2. SSRT Results

**Figure 7:**
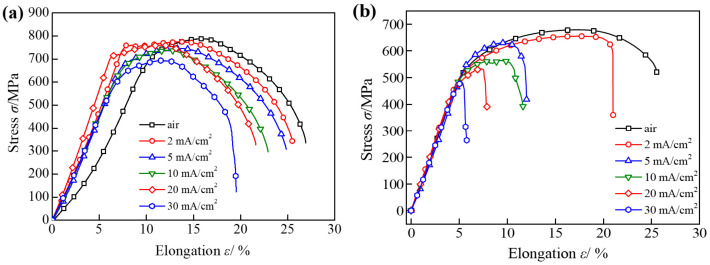
SSRT curves of the X100 steel and welded joint: (**a**) stress–strain curves of the base metal, (**b**) stress–strain curves of the welded joint.

**Figure 8:**
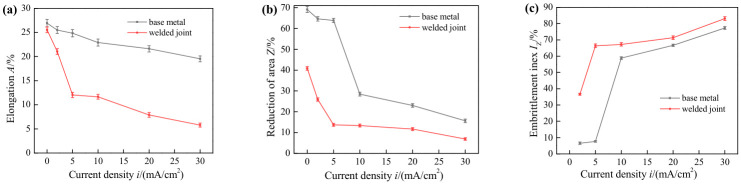
Effect of current density on (**a**) elongation, (**b**) reduction of area and (**c**) embrittlement index.

The SSRT curves of the X100 steel and its welded joint under different hydrogen charging current densities are illustrated in Figure 7. Figure 8 and Table 3 showed the effect of current density on elongation, reduction of area, and HE index, as well as the standard deviations in mechanical properties. The HE indexes of the base metal and welded joint increased with increasing current density, and *I*_ZW_ was higher than *I*_ZB_ under the same current density, showing a higher HIC sensitivity of the welded joint compared to the base metal. The welded joint was observed to be quite susceptive to HIC, with the elongation and reduction in area reducing by 9.01% and 12.17%, respectively, even in the low current density range varying from 2 mA/cm^2^ to 5 mA/cm^2^. When the current density was 2 mA/cm^2^, The HE index of the welded joint was 36.6%, which was higher than the critical value of the HE index of the evaluation standard (35%) [6], implying a high HIC susceptibility. However, the HE index of the base metal was relatively low with the current density lower than 5 mA/cm^2^, and then increased quickly with the current density varying from 5 mA/cm^2^ to 10 mA/cm^2^. The HE indexes of the base metal and the welded joint both increased relatively slowly under the current density varying from 10 mA/cm^2^ to 30 mA/cm^2^.

**Figure 9:**
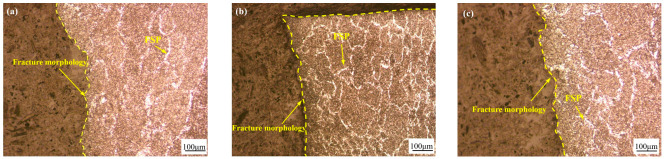
Fracture surfaces of welded joint SSRT specimens under different hydrogen charging current densities: (**a**) 2 mA/cm^2^ (**b**) 5 mA/cm^2^ (**c**) 30 mA/cm^2^.

**Figure 10:**
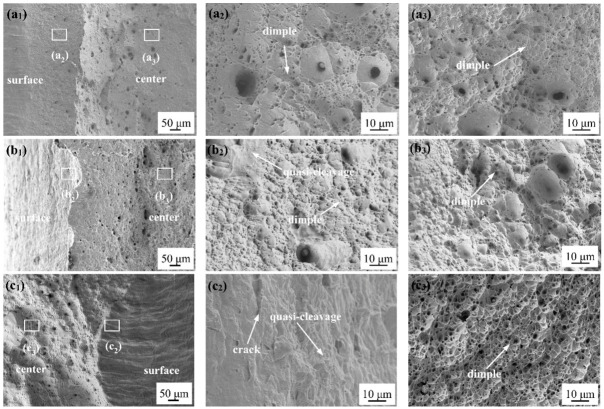
Fracture surface morphologies in the BM SSRT specimens: (**a1**–**a3**) overall morphology, detail of area a2, detail of area a3 in the specimen with current density of 2 mA/cm^2^, (**b1**–**b3**) overall morphology, detail of area b2, detail of area b3 in the specimen with current density of 10 mA/cm^2^, (**c1**–**c3**) overall morphology, detail of area c2, detail of area c3 in the specimen with current density of 30 mA/cm^2^.

**Figure 11:**
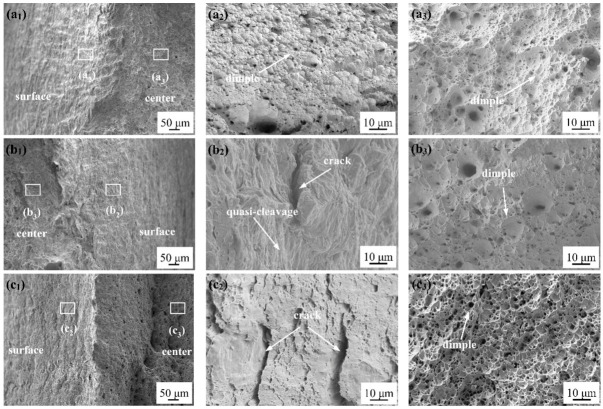
Fracture morphologies in the welded joint SSRT specimens: (**a1**–**a3**) overall morphology, detail of area a2, detail of area a3 in the specimen with current density of 2 mA/cm^2^, (**b1**–**b3**) overall morphology, detail of area b2, detail of area b3 in the specimen with current density of 10 mA/cm^2^, (**c1**–**c3**) overall morphology, detail of area c2, detail of area c3 in the specimen with current density of 30 mA/cm^2^.

The fracture positions in the welded joint specimens were analyzed and found to be in the weld metal (Figure 9). It can be seen that the microstructure near the fracture surface include FSP, which is a typical microstructure in the weld metal, showing that the welded joint SSRT samples fractured in the WM. The fracture surfaces of all SSRT specimens were observed by SEM and are illustrated in Figure 10 and Figure 11. Figure 10(a1,b1,c1) and Figure 11(a1,b1,c1) refer to the macro-fracture surface morphologies of the BM samples and welded joint samples. Figure 10(a2,b2,c2) and Figure 11(a2,b2,c2) refer to the near-surface fracture morphologies of the BM samples and welded joint samples, while Figure 10(a3,b3,c3) and Figure 11(a3,b3,c3) refer to the fracture morphologies in the central zones. The near-surface fracture morphologies of the BM samples and welded joint samples were predominantly ductile dimples pattern under a hydrogen charging current density of 2 mA/cm^2^ (Figure 10(a2) and Figure 11(a2)). The larger dimples were surrounded by some smaller dimples, indicating typically micro-void coalescence (MVC) fracture mode. The number of dimples in the welded joint samples was smaller than that in the BM samples. As the current density increased to 10 mA/cm^2^, the near-surface fracture morphologies of the BM and welded joint samples showed fewer dimples with finer sizes and depths, and more river pattern lines appeared, suggesting the fracture mode changed from ductile MVC to quasi-cleavage fracture mode (Figure 10(b2) and Figure 11(b2)). When the hydrogen charging current density was 30 mA/cm^2^, the subsurface fracture morphologies of the BM and welded joint samples exhibited typical brittle fracture characteristics (Figure 10(c2) and Figure 11(c2)), and more longer cracks can be seen in the welded joint. Ductile morphologies of dimples were the main characteristics of the central zones for both BM and welded joint samples (Figure 10(a3,b3,c3) and Figure 11(a3,b3,c3)). As the current density increased, the number of dimples gradually decreased. This fracture morphology change trend validates that the plasticity of samples degraded and the HE indexes elevated. The subsurface zones were more brittle than the central zones for all the samples. Since hydrogen atoms entered the material from the surface and then diffused into the interior, the hydrogen concentration in the zones decreased in the following order: surface, subsurface and central zone. Therefore, only the surface and subsurface in the samples exhibited embrittlement to some extent, while the embrittlement of the central zones was barely observed for all the samples.

### 3.3. Hydrogen Permeation Test Results

**Figure 12:**
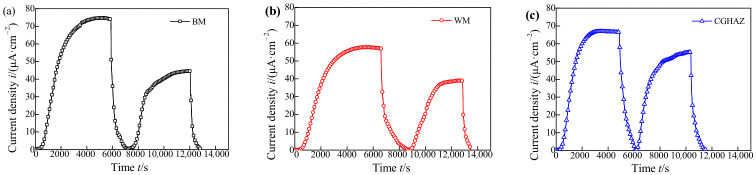
Electrochemical hydrogen permeation curves of (**a**) BM, (**b**) WM and (**c**) CGHAZ.

The two-cycle hydrogen permeation curves of the BM, WM, and CGHAZ are shown in Figure 12. Hydrogen permeation parameters of the BM, WM, and CGHAZ based on Figure 12 are shown in Table 4. In the first cycle, a large number of irreversible and reversible hydrogen traps had to be completely filled before hydrogen atoms could diffuse to the anode and form the anode current. In the second hydrogen permeation cycle, the irreversible hydrogen traps had already been filled, and the hydrogen atoms in the irreversible hydrogen traps did not have sufficient energy to separate from the traps, losing their diffusion ability. Only the reversible hydrogen traps inside the sample were responsible for hydrogen trapping, inducing shorter penetration times for the second cycles.

*D*_eff_ value of the WM (5.38 × 10^−5^ cm^2^·s^−1^) was smaller than that of the BM (6.23 × 10^−5^ cm^2^·s^−1^) and CGHAZ (8.26 × 10^−5^ cm^2^·s^−1^), while *N*_T_ of the WM (4.09 × 10^17^ cm^−3^) was higher than that of the BM (3.49 × 10^17^ cm^−3^) and CGHAZ (1.23 × 10^17^ cm^−3^). The low hydrogen diffusivity and a great number of trapping sites could induce a high sensitivity to HIC, as confirmed by Saleh and Dong [31,32]. The irreversible hydrogen trap density *N*_ir_ was higher than the reversible hydrogen trap density *N*_r_ for the BM, WM, and CGHAZ, indicating that the irreversible hydrogen traps had a significant impact on the *D*_eff_. The *N*_ir_ and *N*_r_ values of the WM were higher than those of the BM and CGHAZ.

## 4. Discussion

### 4.1. Effect of Microstructure on HIC Susceptibility

The HIC sensitivity of the welded joint is greatly affected by the microstructure, which is a main factor affecting the hydrogen trapping and diffusion process. Bainitic microstructures are sensitive to HIC since bainite lath boundaries and M-A constituents are effective hydrogen traps. Though the inclusions, voids, and M-A constituents can be HIC nucleation sites, HIC cracks are also likely to propagate along bainitic lath interfaces due to serious hydrogen trapping and accumulation [1]. Hydrogen can accumulate along bainite inter-lath surface, inducing inter-lath surface separation and final micro-crack initiation [1,33]. AF microstructure is reported to act as a reversible hydrogen trap site with high-density dislocations, and it is more efficient in trapping hydrogen than bainite. However, AF is less sensitive to HIC due to its high toughness [34]. FSP is reported to be detrimental to the weld metal toughness and HIC resistance due to its large grain size [35].

Another important factor that affects HIC but may not be a decisive factor is the grain size [36]. It is widely accepted that the impact toughness of metals and alloys can be improved by decreasing the grain size. It is also reported that the smaller the grain size, the better resistance of metals to HIC that can be achieved [37]. It is obvious that the grain sizes of the WM and BM were estimated at about 30~80 μm and 10~20 μm, respectively. Due to the coarse microstructure in the WM, fewer hydrogen atoms were trapped in the grain boundaries due to a relative smaller grain boundary area per unit volume. Therefore, more hydrogen can diffuse through the grain boundaries and may accumulate in other irreversible hydrogen traps like second phases and inclusions, thus inducing crack initiation.

The distribution, area fraction, and size of the M-A constituents in the metal also affect the HIC susceptibility. Park [34] reported that the existence of M-A constituents induced high HIC sensitivity since M-A constituents were strong hydrogen trap sites in the microstructures. Based on Figure 2 and Figure 4, the sizes of slender M-A constituents in WM were about 0.2~0.5 μm in width and 1.0~10.0 μm in length, while the sizes of blocky M-A constituents in WM were about 0.5~2.0 μm in width and 1.0~3.0 μm in length. However, M-A constituents in the BM were mostly blocky islands with sizes of 0.4~2.0 μm in length and 0.2~1.8 μm in width, and they were too small to work as effective crack initiation positions. Thus, the average size of M-A constituents in the BM was smaller than that in the WM, and it was relatively small for HIC nucleation. The area fraction of M-A constituents is another effective criterion to evaluate the HIC susceptibility. Based on Table 2, it is seen that the amount of M-A constituents in the WM (11.21%) was larger than that in the BM (7.73%). The higher M-A phase fraction in the WM than the BM was responsible for a higher HIC susceptibility of the WM. When the trapped hydrogen atoms accumulated to a certain amount around the M-A constituents, the M-A/matrix interfacial bonds were weakened and high hydrogen pressure could induce the debonding or crack initiation under a low stress.

The coarse microstructure in the WM was characterized by a certain amount of GB and FSP, which were reported to be sensitive to HIC. Additionally, the WM was also characterized by a large grain size and a high M-A fraction (11.21%), leading to significant plasticity loss and high HE index of 36.6% even under a low current density of 2 mA/cm^2^ (Figure 7). As shown in Figure 2a, the microstructure of the X100 steel was composed of relatively soft FGF and regularly embedded reinforced GB, conforming to a typical dual phase structure [12]. Though the BM microstructure contained 7.73% M-A islands with an average size below 2 μm, these M-A islands were distributed evenly and were too small to initiate cracks [2]. The relative lower HE index of the BM than the WM can be attributed to a fine grain size and even distribution of relative fine M-A constituents.

### 4.2. Effect of Inclusions on HIC Susceptibility

Non-metallic inclusions like oxide inclusions, MnS, TiN, VC, and complex carbon-nitrides belong to irreversible traps [4], and they have been reported to be influential in causing HIC since the inclusions are considered efficient hydrogen traps.

**Figure 13:**
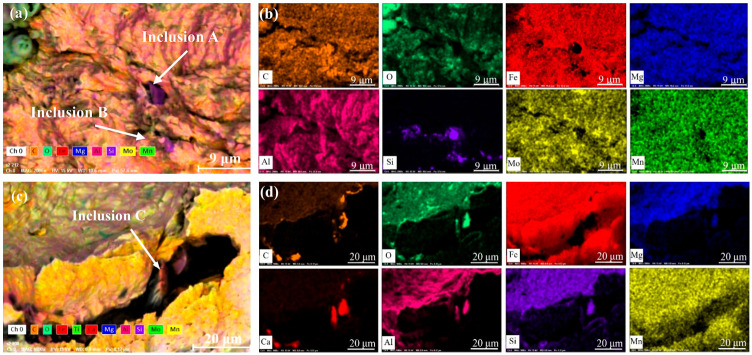
SEM images of HIC cracks in fracture surface: (**a**) inclusion A and B in the cracked zone of the BM, (**b**) EDS maps of A and B in (**a**), (**c**) inclusion C in the cracked zone of the WM, (**d**) EDS maps of inclusion C in (**c**).

Figure 13 shows the HIC cracks in the fracture surface and the EDS maps in the cracked zones. It is evident from Figure 13a–c that the sizes of inclusions A, B, and C were larger than 2 μm, and inclusions A and B in the cracked zone were enriched in Si, indicating Si-enriched inclusions were related to HIC, which was also proved by Jack [15]. Si-enriched inclusions are deformable, and the interfacial spaces between the silicon oxide and metal matrix favor microcrack nucleation. Based on the results shown in Figure 13c,d, it is evident that inclusion C in the cracked zone was enriched in Ca–A–Si–O, indicating Ca–Al–Si–O inclusions were locations of cracking initiation, which was also documented and proved by Saleh [31]. Hydrogen atoms could migrate in the metal and accumulate in micro-voids and inclusion/matrix spaces. Then, hydrogen atoms combined into molecules and aggregated, leading to a pressure high enough to induce crack nucleation [4].

### 4.3. Hydrogen Diffusion Behavior

The calculated values of *D*_eff_ in the WM, CGHAZ, and BM are in the order of 10^−5^ cm^2^/s, which are lower than the hydrogen diffusivity in α-Fe (10^−4^ cm^2^/s) [27]. The hydrogen diffusivity differences can be attributed to the presence of hydrogen traps in the metals. Hydrogen in the welded joint is easily trapped by traps such as non-uniform components and crystal defects (dislocations, vacancies, grain boundaries) and the traps can be classified into two categories in view of the binding energy, namely reversible traps and irreversible traps. Typical reversible hydrogen traps include substitutional atoms, micro voids, dislocations, and grain boundaries, with binding energies below 60 kJ/mol [6]. Typical irreversible traps with binding energies above 60 kJ/mol include interfaces and second phases such as nitrides and oxides, and they can fix hydrogen until saturation is reached. The binding energies of hydrogen traps can be affected by factors like their shape, size, location, etc. [4]. Defects such as grain boundaries and dislocations may not only capture hydrogen as hydrogen traps, but also form channels for hydrogen diffusion [6].

Compared to the CGHAZ and BM, the value of *D*_eff_ in the WM was the lowest. This result is validated by its highest density of hydrogen trap sites. It is acknowledged that hydrogen trapping efficiency increases in the order of ferrite, pearlite, bainite and acicular ferrite [29]. The WM was characterized with a GB + AF + FSP microstructure, a large number of M-A constituents and inclusions which were strong hydrogen traps. Thus, the hydrogen-trapping efficiency in the WM was the highest, with a *N*_T_ value of 4.09 × 10^17^ cm^−3^, inferring more hydrogen trapping sites such as inclusions, dislocations, bainitic lath boundaries, and M-A/matrix interfaces. Furthermore, the BM with small grain size was characterized with fine GB and FGF. The high level of *D*_eff_ in the BM is mainly due to the larger grain boundary area and relative fewer trapping sites compared to the WM. Compared to the BM, the *D*_eff_ value in the CGHAZ was higher, though CGHAZ had a similar inclusion amount and a higher M-A area fraction. Additionally, the CGHAZ exhibited a lower *N*_T_ value than the BM. The reason for the higher *D*_eff_ in the CGHAZ than in the BM is that the fraction of large-angle grain boundaries decreased due to complete austenitization and grain coarsening effects. It is reported by Zhao et al. [38] that large-angle grain boundaries with boundary angles above 15° are more effective in trapping hydrogen than small-angle grain boundaries. Additionally, the dislocation density in the CGHAZ was lower than that in the BM, with high level of dislocation density generated during the thermal–mechanical-controlled process.

Furthermore, the smaller *D*_eff_ and higher *N*_T_ of the WM than the BM indicates a higher HIC susceptibility of the WM than the BM. Thus, the relatively high HIC susceptibility of the WM can be attributed to a mainly bainitic microstructure, with large numbers of M-A constituents and inclusions. The results are generally in agreement with the SSRT results presented above.

## 5. Conclusions

The main conclusions are drawn from the studies above and listed below. These findings are specific to the tested steel and welding method.

(1) The HIC susceptibility of X100 steel welded joint using SMAW was investigated. The SSRT test results show that HE indexes of the BM and welded joint both increased with increasing current density, and the welded joint exhibited a higher HE index compared to the BM under the same current density.

(2) The degradation of the strength and plasticity with increasing charging current density was also associated with the fracture mode, from MVC ductile fracture mode to a brittle fracture mode for both the BM and the welded joint.

(3) The content of inclusions with sizes larger than 2 μm was 24.92% in the WM, higher than that in the BM (12.73%). The HIC cracks were mainly caused by the existence of Si-enriched inclusions for both the BM and the welded joint. There was no HIC crack found to be associated with inclusions enriched in MnS.

(4) The sizes of the slender and blocky M-A constituents in the WM were 1.0~10.0 μm and 1.0~3.0 μm in length, 0.2~0.5 μm and 0.5 ~2.0 μm in width, respectively. The sizes of M-A constituents in the BM were smaller than 2 μm, which was too small to initiate cracking. The M-A constituent fraction in the WM was 1.5 times that in the BM. The average grain size in the WM (78.3 μm) was larger than that in the BM (11.7 μm). The large number of M-A constituents and large grain size in the WM were also responsible for a higher HIC susceptibility of the WM than the BM.

## Figures and Tables

**Table 1:** Chemical composition of base material and electrode (wt.%).

Materials	C	Mn	Si	P	S	Cr	Nb	Mo	Ni	Cu	Fe
X100 steel	0.050	1.950	0.260	0.011	0.003	0.026	0.048	0.240	0.360	0.200	balance
Electrode	0.050	1.580	0.410	0.008	0.005	0.380	0.001	0.360	1.820	0.200	balance

**Table 2:** Statistical results of the M-A constituent fractions in different subzones (%).

Location	BM	WM	CGHAZ	FGHAZ
Content (%)	7.73	11.21	10.52	9.47
Standard error	0.0426	0.0392	0.0359	0.0404

**Table 3:** SSRT test results with different charging current densities.

Condition	Base Metal			Welded Joint		
	Elongation *A*/%	Reduction in Area *Z*/%	HE Index *I*_ZB_/%	Elongation *A*/%	Reduction in Area *Z*/%	HE Index *I*_ZW_/%
In air	26.92 ± 0.78	69.16 ± 1.44	—	25.54 ± 0.60	40.85 ± 0.85	—
2 mA/cm^2^	25.49 ± 0.73	64.64 ± 1.05	6.54 ± 0.75	21.03 ± 0.60	25.90 ± 0.77	36.60 ± 0.58
5 mA/cm^2^	24.84 ± 0.74	63.88 ± 1.04	7.63 ± 0.50	12.02 ± 0.53	13.73 ± 0.69	66.39 ± 1.07
10 mA/cm^2^	22.91 ± 0.73	28.47 ± 0.92	58.83 ± 0.80	11.65 ± 0.50	13.38 ± 0.67	67.25 ± 1.05
20 mA/cm^2^	21.61 ± 0.66	23.05 ± 0.90	66.67 ± 0.73	7.90 ± 0.50	11.70 ± 0.71	71.36 ± 1.13
30 mA/cm^2^	19.53 ± 0.61	15.67 ± 0.85	77.34 ± 0.84	5.79 ± 0.40	6.90 ± 0.61	83.11 ± 1.14

**Table 4:** Parameters of hydrogen permeation test for different subzones in the welded joint.

Specimens	$J_{\infty }$ mol·cm^−2^·s^−1^ × 10^−10^	*D*_eff_ cm^2^·s^−1^ × 10^−5^	*C*_0_ mol·cm^−3^ × 10^−6^	*N*_T_ cm^−3^ × 10^17^	*N*_r_ cm^−3^ × 10^16^	*N*_ir_ cm^−3^ × 10^17^
BM	7.75	6.23	9.95	3.49	7.10	2.78
WM	5.98	5.38	8.90	4.09	9.36	3.16
CGHAZ	6.94	8.26	6.73	1.23	1.89	1.04

## Data Availability

The original contributions presented in this study are included in the article. Further inquiries can be directed to the corresponding author.
